# In dogs with gastric dilatation volvulus (GDV) undergoing gastropexy, what is the rate of recurrence of GDV?

**DOI:** 10.18849/ve.v10i2.709

**Published:** 2025-05-14

**Authors:** Daniel Low

**Keywords:** canine, dog, emergency surgery, gastric dilatation volvulus, gastropexy, soft tissue surgery

## Abstract

**PICO question:**

In dogs with a gastric dilatation volvulus (GDV) treated surgically and undergoing a gastropexy, what is the rate of recurrence of GDV?

**Clinical bottom line:**

**Category of research:**

Risk.

**Number and type of study designs reviewed:**

Sixteen studies were critically appraised. Two were prospective randomised controlled trials, two were prospective cohort studies, one was a case-control study, and eleven were case series.

**Strength of evidence:**

Weak.

**Outcomes reported:**

A low rate of recurrence of GDV after gastropexy was consistently reported, although methodological limitations were recognised in all studies.

**Conclusion:**

Overall, the studies provide weak evidence that the rate of recurrent GDV is low after gastropexy.

## How to apply this evidence in practice

The application of evidence into practice should take into account multiple factors, not limited to: individual clinical expertise, patient’s circumstances and owners’ values, country, location or clinic where you work, the individual case in front of you, the availability of therapies and resources.

Knowledge Summaries are a resource to help reinforce or inform decision-making. They do not override the responsibility or judgement of the practitioner to do what is best for the animal in their care.

## Clinical scenario

You are about to operate on a 3-year-old German Shepherd presented with gastric dilatation volvulus (GDV), and you recommend a gastropexy at the same time, as you and other colleagues regularly recommend. The client asks “even with a gastropexy, what are the chances this could happen again?”

## The evidence

Sixteen studies were identified that were relevant to the PICO question. Two prospective clinical trials (Eggertsdóttir et al., 1996; Eggertsdóttir et al., 2001) were appraised. Fourteen observational studies (Belandria et al., 2009; Belch et al., 2017; Benitez et al., 2013; Formaggini & Degna, 2018; Funkquist, 1979; Glickman et al., 1998, Jennings & Butzin, 1992; Leib et al., 1985; Mann et al., 2023; Meyer-Lindenburg et al., 1993; Przywara et al., 2014; Rawlings et al., 2002; Ullmann et al., 2015; Wacker et al., 1998) were appraised and in addition to limitations inherent to the nature of the study, minor limitations in methodologies were recognised. Overall, this body of evidence is of low quality given the study designs of the included papers.

## Summary of the evidence

**Belandria et al. (2009):** Gastropexy with an automatic stapling instrument for the treatment of gastric dilatation and volvulus in 20 dogs **Aim: **To describe the results of long-term follow-up evaluation of 20 dogs in which a stapled gastropexy was performed as a part of the treatment of GDV.

Population	Dogs presented to the emergency rooms of one of two veterinary hospitals in the United States during an unspecified time period with gastric dilatation volvulus (GDV), based on their clinical history and physical examination.
Sample Size	20 dogs.
Intervention Details	The long-term outcome of 20 dogs who had undergone surgical treatment for GDV followed by a staple gastropexy with a gastrointestinal anastomosis stapling device was reported. Long-term follow-up was made initially via telephone and then with physical exam and imaging, where possible. Methodology was not reported and nature (prospective or retrospective) could not be determined. 2/20 dogs had concurrent splenectomy, 2/20 dogs had concurrent gastrotomy, 1/20 dogs had concurrent partial gastrectomy.
Study design	Dual-centre case series.
Outcome studied	Long-term outcome and recurrence of GDV.
Main findings (relevant to PICO question)	19/20 survived to discharge. 2/19 were lost to long-term follow-up. 17/19 had long-term telephone follow-up available, mean follow-up time was 20.9 months (range 5 to 43 months). 14/17 dogs were alive at the time of telephone follow-up. 3/17 dogs died or were euthanised for unrelated reasons. 11/14 dogs had physical exam, radiographic, and ultrasonographic follow-up available, mean follow-up time was 22 months (range 16 to 42 months). 11/11 dogs showed imaging evidence of intact gastropexies. 0/17 dogs had recurrence of GDV.
Limitations	Criteria for case selection was not reported, other inclusion and exclusion criteria were also not reported. Due to limitations of follow-up methodology, recurrent GDV could not be conclusively excluded in 5/19 dogs as 2/19 dogs did not receive long term follow up, and 3/17 dogs did not receive physical exam, radiographic or ultrasonographic follow up. No mention of statistical analysis being performed. Only employed one method of gastropexy. Absence of a control group. Length of follow-up was limited with less than 12-month follow-up available in two dogs and lack of systematic lifetime follow-up to definitively exclude recurrent GDV. Genetic and lifestyle bias of sample population from a limited geographical area. Referral hospital bias.

Table note...

**Belch et al. (2017):** Modified tube gastropexy using a mushroom-tipped silicone catheter for management of gastric dilatation-volvulus in dogs **Aim: **To describe the surgical technique and to report the short- and long-term complications and clinical outcomes of a retrospective cohort of dogs managed for GDV, using a modified right-sided tube gastropexy technique, combining tube and incisional gastropexies, using a mushroom-tipped silicone catheter.

Population	Dogs presenting to a multidisciplinary specialist referral centre in the United Kingdom between January 2007 and December 2014, with a diagnosis of gastric dilatation volvulus (GDV).
Sample Size	36 dogs.
Intervention Details	Case records were reviewed for dogs who had undergone surgical treatment for GDV and who had an open approach to modified tube gastropexy. Long-term follow-up was conducted remotely through contact with referring veterinarians. 31/36 dogs were surgically treated for GDV with a modified tube gastropexy. 5/36 had another gastropexy technique and were excluded.
Study design	Retrospective single-centre case series.
Outcome studied	Long-term outcome and recurrence of GDV.
Main Findings (relevant to PICO question)	30/31 dogs survived to discharge. 21/30 had long-term remote follow-up available, median follow-up time was 2.5 years (range 3 months to 5 years). 19/21 dogs were alive at the time of telephone follow-up. 2/21 dogs died or were euthanised for unrelated reasons. 0/21 dogs had recurrence of GDV.
Limitations	The study excluded 5/36 dogs as they received a different gastropexy technique. No diagnostic imaging was used to determine the gastropexies were intact. Not all records were complete. Absence of a control group. Criteria for selecting a modified gastrostomy tube or another gastropexy technique was not reported. Due to limitations of follow-up methodology, recurrent GDV could not be conclusively excluded in 1/2 dogs who died and impacts certainty of study results. Limitations of length of follow-up was limited with less than 12-month follow-up available in five dogs and lack of systematic lifetime follow-up to definitively exclude recurrent GDV. Genetic and lifestyle bias of sample population from a limited geographical area. Referral hospital bias.

Table note...

**Benitez et al. (2013):** Efficacy of Incisional Gastropexy for Prevention of GDV in Dogs **Aim: **To evaluate the efficacy of incisional gastropexy performed either during the surgical treatment of GDV or as a prophylactic measure.

Population	Dogs presenting to a university teaching hospital in the United States between 2000 and 2010, both with and without gastric dilatation volvulus (GDV), and who underwent an incisional gastropexy.
Sample Size	61 dogs.
Intervention Details	Case records were reviewed for dogs who had undergone surgical treatment for GDV and who had an open approach to incisional gastropexy, and who had an open approach to incisional gastropexy as a prophylactic surgery, without prior GDV. Group A: 27/61 dogs had prophylactic gastropexy. Group B: 34/61 dogs had gastropexy at the same time as treatment for a GDV episode. Long-term follow-up was conducted with a combination of remote follow-up (method unspecified), physical examination, diagnostic imaging, and necropsy.
Study design	Retrospective single-centre case series.
Outcome studied	Long-term outcome and recurrence of GDV.
Main Findings (relevant to PICO question)	Long-term follow-up was available in 61 dogs. Median follow-up time for both groups was 717 days (range 49 to 2511 days). Median follow-up time for the prophylactic gastropexy group was 581 days (range 53 to 2511 days). Median follow-up time for the gastropexy after a GDV group was not reported. 31/61 dogs were alive at the time of follow-up. 30/61 dogs died or were euthanised for unrelated reasons. 3/30 dogs who died or were euthanised for unrelated reasons had necropsy evidence of an intact gastropexy. 0/61 dogs had a subsequent episode of GDV.
Limitations	Retrospective nature. Analysis included both prophylactic gastropexies and gastropexies at the time of treatment of a GDV. Only one gastropexy method was studied. Intact gastropexy was only confirmed in the 3 dogs that received post-mortems. No imaging performed to confirm intact gastropexies. Due to limitations of remote follow-up, recurrent GDV could not be conclusively excluded in the dogs who died or were euthanised and impacts certainty of study results. Dropout bias, the rate of successful long-term owner contact was not reported. Genetic and lifestyle bias of sample population from a limited geographical area. Referral hospital bias. Length of follow-up was limited with lack of systematic lifetime follow-up to definitively exclude recurrent GDV.

Table note...

**Eggertsdóttir et al. (1996):** Comparison of Two Surgical Treatments of Gastric Dilatation-Volvulus in Dogs **Aim: **To compare the results of GDV correction without gastropexy and GDV correction with gastropexy.

Population	Dogs presented to a university hospital in Norway between March 1991 and November 1992, with a diagnosis of GDV.
Sample Size	36 dogs.
Intervention Details	Group A: open approach to treatment of GDV followed by gastric body biopsy and modified circumcostal gastropexy (n = 21). Group B: open approach to treatment of GDV followed by gastric body biopsy without gastropexy (n = 10). Follow-up was performed 3 times in the first 12 months via physical examination. Follow-up was performed non-systematically thereafter and method of follow-up was not reported. 36 dogs were surgically treated for GDV. 5/36 were excluded due to not surviving to discharge. 31 dogs surgically treated for GDV underwent randomisation, 21/31 dogs were assigned to Group A and 10/31 dogs were assigned to Group B. 2/31 dogs were euthanised intraoperatively.
Study design	Prospective single-centre non-blinded non-randomised controlled trial.
Outcome studied	Long-term outcome and recurrence of GDV.
Main Findings (relevant to PICO question)	5/31 dogs died 4 to 18 days after surgery. Group A: 16/21 dogs had follow-up available, median (range 183 to 918 days) and mean follow-up time was 397 and 430 days respectively. 10/16 dogs were alive at the time of follow-up. 6/16 dogs had died or were euthanised due to unrelated reasons, 0/10 dogs had recurrence of GDV. Group B: 8/10 dogs had follow-up available, median (range 30 to 898 days) and mean follow-up time was 106 and 228 days respectively. 2/8 dogs were alive at the time of follow-up, 3/8 dogs had died or were euthanised due to unrelated reasons, 3/8 dogs experienced a recurrence of GDV. Death from recurrent GDV or related causes were 3/16 dogs (19%) and 5/7 dogs (71%) for Group A and B respectively (P = 0.02). Median survival time was 549 days and 107 days for Group A and B respectively (P = 0.04).
Limitations	Selection bias of study participants in that only dogs with no history of GDV in the last 5 months were eligible for study inclusion. Enrollment bias in a prospective trial. Major limitations in randomisation methodology: unbalanced (2:1) randomisation, demographic data of groups not statistically compared, Group B was clinically more unstable than Group A. Dropout bias with 6 dogs dying due to non-GDV related causes and 2 dogs lost to follow-up. Genetic and lifestyle bias of sample population from a limited geographical area. Referral hospital bias. Length of follow-up was limited with lack of systematic lifetime follow-up to definitively exclude recurrent GDV.

Table note...

**Eggertsdóttir et al. (2001):** Comparison of the Recurrence Rate of Gastric Dilatation With or Without Volvulus in Dogs After Circumcostal Gastropexy Versus Gastrocolopexy **Aim: **To compare the recurrence rate of acute gastric dilatation with or without volvulus (GDV) after circumcostal gastropexy (CCGP) or gastrocolopexy (GCP) in dogs.

Population	Dogs presented to two university hospitals in Norway and Denmark between February 1996 and July 1998, with a diagnosis of gastric dilatation volvulus (GDV).
Sample Size	54 dogs.
Intervention Details	54 dogs surgically treated for GDV underwent randomisation. Group A: open approach to treatment of GDV followed by modified circumcostal gastropexy (n = 27). Group B: open approach to treatment of GDV followed by gastrocolopexy, where a 10–15cm pexy was created along the transverse colon and the greater curvature of the stomach, just ventral to the attachment of the greater omentum (n = 27). 3 dogs in Group A and 2 dogs in Group B were euthanised intraoperatively. 2 dogs in Group A and 5 dogs in Group B died within 180 days from causes other than recurrent GDV. Follow-up was performed 3 times in the first 12 months via physical examination. Follow-up was performed non-systematically thereafter and method of follow-up was not reported.
Study design	Prospective dual-centre non-blinded randomised controlled trial.
Outcome studied	Long-term outcome and recurrence of GDV.
Main Findings (relevant to PICO question)	Group A: 22/27 dogs had long-term follow-up available, median follow-up time was 700 days, mean >600 days, ranges not reported. 14/22 dogs were alive at the time of follow-up. Group B: 20/27 dogs had long-term follow-up available, median follow-up time was 400 days, mean >500 days, ranges not reported. 13/20 dogs were alive at the time of follow-up. 1/13 survivor dogs were reported to have had a recurrence of GDV, confirmed surgically and addressed with circumcostal gastropexy. Rate of recurrent GDV was 2/22 (9%) and 4/20 (20%) for Group A and B respectively (P = 0.4). Median survival time was 549 days and 107 days for Group A and B respectively (P = 0.04).
Limitations	Recruitment and selection bias of study participants. Enrollment bias in a prospective trial. Major limitations in blinding of trial and limited details. Demographic data of groups not statistically compared and groups were reported to be equivalent without evidence. Limitations in definition of GDV recurrence. No necropsy or imaging evidence was available to support that 1/8 of the dogs died of recurrent GDV from Group A, or that that 1/7 of the dogs died of recurrent GDV from Group B. Only two gastropexy methods were studied. Absence of a control group. Dropout bias in that 7 dogs in Group A and 4 dogs in Group B died from causes other than recurrent GDV. Due to limitations of remote follow-up, recurrent GDV could not be conclusively excluded in 12/16 dogs who died or were euthanised and recurrent GDV could not be conclusively diagnosed in 1/3 dogs. Genetic and lifestyle bias of sample population from a limited geographical area. Referral hospital bias.

Table note...

**Formaggini & Degna (2018):** A Prospective Evaluation of a Modified Belt-Loop Gastropexy in 100 Dogs with Gastric Dilatation-Volvulus **Aim: **To describe a modified belt-loop gastropexy and determine its intraoperative complications and long-term efficacy.

Population	Dogs presented to a veterinary hospital in Italy during an undefined 3-year period, with a diagnosis of gastric dilatation volvulus (GDV) and who underwent surgery.
Sample Size	110 dogs.
Intervention Details	Cases prospectively recruited to undergo an open approach to a modified belt-loop gastropexy. 110 dogs were surgically treated for GDV and had modified belt-loop gastropexy. 3/110 dogs died intraoperatively. Minimum of 1-year telephone follow-up for study inclusion. Necropsy where available.
Study design	Prospective single-centre case series.
Outcome studied	Long-term outcome and recurrence of GDV.
Main Findings (relevant to PICO question)	7/110 dogs were lost to follow-up. 11/100 dogs died ≤ 5 days after surgery. 1/100 dogs died 3 months after surgery for unrelated reasons. 10/100 dogs died ≤ 1 year after surgery due to unknown causes but not including recurrent GDV, no necropsy or imaging evidence was available. 78/110 dogs had follow-up available at a median (range 450–1200 days) and mean of 850 days and 709. 0/78 dogs had recurrence of GDV.
Limitations	Selection bias. Dropout bias. Due to limitations of remote follow-up, recurrent GDV could not be conclusively excluded in 10/10 dogs who died or were euthanised. No necropsy or imaging evidence was available to support that 0/78 dogs had recurrence of GDV. Absence of a control group. Genetic and lifestyle bias of sample population from a limited geographical area.

Table note...

**Funkquist (1979):** Gastric torsion in the dog III. Fundic gastropexy as a relapse-preventing procedure **Aim: **To describe the outcome of dogs undergoing fundic gastropexy.

Population	Dogs presented between 1 January 1974 and 30 June 1978, with a diagnosis of gastric dilatation volvulus (GDV) and who underwent staged treatment of GDV and fundupexy.
Sample Size	36 dogs.
Intervention Details	Open approach to left fundupexy performed 1 to 14 weeks after an episode of GDV.
Study design	Retrospective case series.
Outcome studied	Long-term outcome and recurrence of GDV, obtained remotely.
Main Findings (relevant to PICO question)	36 dogs underwent left fundupexy. 1/36 died 8 days after fundupexy for unrelated reasons. 1/36 was lost to follow-up. 34/36 dogs had long-term follow-up available with mean follow-up time of 18 months (range 1.5 months to 4.5 years). 6/34 dogs died or were euthanised for unrelated reasons, necropsy available in all 6 dogs and a failed fundupexy was found in 1 dog. 0/34 dogs had recurrence of GDV.
Limitations	Selection bias. Limited description of methodology. Due to limitations of follow-up, recurrent GDV could not be conclusively excluded in 1 dog who lost to follow-up. Length of follow-up was limited with lack of systematic lifetime follow-up to definitively exclude recurrent GDV.

Table note...

**Glickman et al. (1998):** A Prospective Study of Survival and Recurrence Following the Acute Gastric Dilatation-Volvulus Syndrome in 136 Dogs **Aim: **To identify the short- and long-term prognostic factors for dogs with the GDV syndrome.

Population	Dogs presented to 27 veterinary clinics in the United States between 1991 and an undefined end point, with a diagnosis of gastric dilatation volvulus (GDV) and who underwent surgery.
Sample Size	136 dogs.
Intervention Details	Group A: dogs undergoing surgery for GDV with gastropexy (n = 74). Of these, dogs had incisional gastropexy (n = 28), circumcostal gastropexy (n = 11), appositional gastropexy (n = 7), tube gastropexy (n = 4), other gastropexies (n = 19), and unspecified gastropexies (n = 11). Group B: dogs undergoing surgery for GDV without gastropexy (n = 11). Long-term survival data was obtained remotely via primary veterinarian and client follow-up.
Study design	Prospective multi-centre observational cohort study.
Outcome studied	Long-term outcome and recurrence of GDV.
Main Findings (relevant to PICO question)	103/136 dogs survived ≥ 7 days. 85/103 dogs had long-term follow-up with a median (range 13 to 1170 days) and mean of 461 days and 471 days. 3/74 dogs from Group B with long-term follow-up developed recurrent GDV. 6/11 dogs from Group A with long-term follow-up developed recurrent GDV. Median survival time was 547 days versus 188 days for gastropexy versus no gastropexy (P = 0.0001).
Limitations	Participation bias and follow-up bias. Non-randomised intervention. Dropout bias. No mention of how recurrent GDV was determined. Selection bias. Genetic and lifestyle bias of sample population from a limited geographical area. Recurrent GDV was not ruled out in the 33/136 dogs who survived < 7 days. Length of follow-up was limited with lack of systematic lifetime follow-up to definitively exclude recurrent GDV.

Table note...

**Jennings & Butzin (1992):** Epidemiology of Gastric Dilatation-Volvulus in the Military Working Dog Program

Population	Military working dogs working in United States military bases worldwide, who have had an episode of gastric dilatation volvulus (GDV).
Sample Size	38 dogs.
Intervention Details	Case records of military working dog deaths from 1 January 1987 to 31 December 1989 were reviewed and dogs who died from GDV were identified. Control group (n = 31) were identified via case record search of military working dog deaths from 1 January 1987 to 31 December 1989. These dogs died due to GDV, none underwent surgery to correct GDV and therefore none underwent a gastropexy. Case group (n = 7) were identified via mail survey during the same period. These dogs had GDV and were treated with surgery (tube gastrostomy, circumcostal gastropexy, incisional gastropexy).
Study design	Retrospective observational case control study.
Outcome studied	Long-term outcome and recurrence of GDV.
Main Findings (relevant to PICO question)	1/7 dogs (14.3%) died of recurrent GDV. This dog had an incisional gastropexy and necropsy confirmed gastropexy breakdown.
Limitations	Selection bias between controls and cases. Population bias of military working dogs with specific signalment, husbandry, and access to veterinary care. Time to follow-up was not defined. Response bias for mail survey. No statistical analysis performed.

Table note...

**Leib et al. (1985):** Circumcostal gastropexy for preventing recurrence of gastric dilatation-volvulus in the dog: An evaluation of 30 cases **Aim: **To evaluate circumcostal gastropexy in clinical patients with regard to recurrence of GDV and integrity of the gastropexy site.

Population	Dogs presented to a university teaching hospital in the United States during an unspecified time period, with a diagnosis of gastric dilatation volvulus (GDV) and who underwent surgery and gastropexy.
Sample Size	30 dogs.
Intervention Details	Case records (n = 30) were reviewed for dogs who had undergone surgical treatment for GDV and who had an open approach to circumcostal gastropexy. Long-term follow-up was performed at 3, 6, 12 months, and then yearly thereafter (end date unspecified), with a combination of questionnaire, physical exam, contrast radiography, and necropsy.
Study design	Prospective single-centre case series.
Outcome studied	Long-term outcome and recurrence of GDV.
Main Findings (relevant to PICO question)	30 dogs surgically treated for GDV survived to discharge. 1/30 dogs were lost to follow-up (time point unspecified). 29/30 dogs had questionnaire follow-up available at a median (range 2 to 28 months) and mean of 13 months and 13.7 months. 12/29 dogs had contrast radiography available at a median (range not reported) and mean of 12 months and 13.5 months which showed evidence of an intact gastropexy. 6/29 dogs died or were euthanised for unrelated reasons at a median time of 12 months postoperatively, necropsy showed an intact gastropexy in all dogs. 5/29 dogs died or were euthanised because of postoperative complications after GDV at a median of 3 days postoperatively. 0/29 dogs had recurrence of GDV with necropsy evidence of intact gastropexies in all dogs.
Limitations	Survival to discharge bias, inclusion criteria not reported. Dropout bias. Absence of a control group. Genetic and lifestyle bias of sample population from a limited geographical area.

Table note...

**Mann et al. (2023):** Comparison of incisional gastropexy with and without addition of two full-thickness stomach to body wall sutures **Aim: **To compare complications between a modified incisional gastropexy (MIG) technique and standard incisional gastropexy (SIG).

Population	Dogs presented to University of Missouri Veterinary Health Centerin the United States between March 2005 and April 2019, who underwent incisional gastropexy.
Sample Size	107 dogs.
Intervention Details	Case records were reviewed for dogs who had a diagnosis of GDV and who had surgical treatment and gastropexy with either a standard incisional gastropexy (SIG) (n = 91) or modified incisional gastropexy (MIG) (n = 16), where two simple interrupted sutures are added cranially and caudally to the gastropexy line, passing full thickness through the stomach wall. Follow-up was performed remotely via telephone or email to the referring veterinarian or pet owner, with short-term follow-up defined as suture removal and long-term follow-up defined as that beyond suture removal.
Study design	Retrospective single-centre case series.
Outcome studied	Long-term outcome and recurrence of GDV.
Main Findings (relevant to PICO question)	Short-term follow-up data was available for 40 dogs (29 SIG, 11 MIG). Long-term follow-up data was available for 14 dogs (5 SIG, 9 MIG). 0/107 dogs with varying follow-up duration data had recurrence of GDV.
Limitations	Treatment bias, in that case selection was non-randomised. Dropout bias, and differing rates of dropout amongst SIG and MIG groups. Limitations of follow-up methodology, recurrent GDV could not be conclusively excluded in the 93 dogs with absence of follow-up data and could not be conclusively excluded in the 14 dogs with long-term follow-up due to limitations of owner and referring veterinarian reporting. Genetic and lifestyle bias of sample population from a limited geographical area.

Table note...

**Meyer-Lindenburg et al. (1993):** Treatment of gastric dilatation-volvulus and a rapid method for prevention of relapse in dogs: 134 cases (1988-1991)

Population	Dogs presented to a clinic in Germany between January 1988 and April 1991, with a diagnosis of gastric dilatation volvulus (GDV).
Sample Size	134 dogs.
Intervention Details	Outcome of dogs with GDV which were managed medically (n = 33) were compared to dogs which had surgical correction of GDV with ventral midline gastropexy (n = 87). Follow-up was performed remotely or physically.
Study design	Retrospective single-centre cohort study.
Outcome studied	Outcome and recurrence of GDV.
Main Findings (relevant to PICO question)	134 dogs presented for GDV. 13/134 dogs died or were euthanised before treatment. 33/121 dogs were successfully managed medically (controls). 25/33 dogs managed medically survived to discharge. 19/25 dogs managed medically had recurrence of GDV, time to follow-up was not reported. 88/121 dogs underwent surgery (cases). 1/88 dogs underwent surgery without gastropexy, recurrence of GDV occurred 1 day after surgery. 87/88 dogs underwent surgery and gastropexy. 63/88 dogs surgically treated survived to discharge. 61/63 dogs surgically treated had long-term follow-up available. 4/61 dogs surgically treated had recurrence of GDV, based on owner reporting. Recurrence rate between cases and controls was significantly different (P = 0.001).
Limitations	Radiographic confirmation of GDV was not performed in all cases. Time to follow-up and range of follow-up was not reported. Inclusion bias, gastric dilatation cases were likely included in sample population along with GDV cases. Treatment bias, only cases which failed stomach tube passage received surgery. Survival to discharge bias, inclusion criteria not reported. Dropout bias. Due to limitations of follow-up methodology, recurrent GDV could not be conclusively confirmed or excluded in 61 dogs. Genetic and lifestyle bias of sample population from a limited geographical area.

Table note...

**Przywara et al. (2014):** Occurrence and recurrence of gastric dilatation with or without volvulus after incisional gastropexy **Aim: **To report GD and GDV recurrence rates after incisional gastropexy.

Population	Dogs presented to a referral centre in the United States between 2004 and 2012, and underwent surgical correction of gastric dilatation volvulus (GDV) followed by gastropexy.
Sample Size	64 dogs.
Intervention Details	Case records were reviewed for dogs who had undergone surgical treatment for GDV and who had an open approach to incisional gastropexy. Follow-up was performed via telephone contact with dog owners at a median (range 2.0 to 8.3 years) and mean follow-up time of 3.9 years and 3.9 years respectively.
Study design	Retrospective single-centre case series.
Outcome studied	Outcome and recurrence of GDV.
Main Findings (relevant to PICO question)	60/64 dogs survived to discharge. 22/60 dogs were alive at the time of follow-up, median (range 2.0 years to 8.3 years) and mean follow-up time was 3.9 years and 3.9 years. 38/60 dogs died or were euthanised for unrelated reasons. 0/60 dogs had a recurrence of GDV.
Limitations	Retrospective study. Absence of a control group. Not all dogs were exposed in a GDV fertile scenario, it is impossible to know if the non-recurrent GDV was due to the gastropexy (if intact) protection or not. Dropout bias. Due to limitations of follow-up methodology, recurrent GDV could not be conclusively excluded in 38 dogs as study relied on owner reporting. Genetic and lifestyle bias of sample population from a limited geographical area. Referral hospital bias.

Table note...

**Rawlings et al. (2002):** Prospective evaluation of laparoscopic-assisted gastropexy in dogs susceptible to gastric dilatation **Aim: **To determine the usefulness and long-term outcome of laparoscopic-assisted gastropexy in the prevention of GDV in client-owned dogs that were susceptible to gastric dilatation.

Population	Dogs presented to a referral centre in the United States between 2004 and 2012, and underwent surgical correction of gastric dilatation volvulus (GDV) followed by gastropexy.
Sample Size	2 dogs.
Intervention Details	2 dogs underwent laparoscopic surgery for GDV followed by laparoscopic-assisted gastropexy.
Study design	Retrospective single-centre case series.
Outcome studied	Long-term outcome of dogs undergoing prophylactic laparoscopic gastropexy (not relevant to PICO), and reported these two dogs who had laparoscopic surgery to treat GDV and perform gastropexy. Long-term outcome of these relevant dogs was not reported.
Main Findings (relevant to PICO question)	2/2 dogs survived to discharge. No short- or long-term outcome was reported.
Limitations	Only discusses survival and gives no indication of recurrence. Very small sample size. Only one gastropexy method was studied. No follow-up available.

Table note...

**Ullmann et al. (2015):** Gastric dilatation volvulus: a retrospective study of 203 dogs with ventral midline gastropexy **Aim: **To evaluate the recurrence rate of gastric dilatation volvulus and the incidence of complications in subsequent coeliotomies following ventral midline gastropexy.

Population	Dogs presented to a university hospital in Germany between January 2000 and December 2009, and underwent surgical correction of gastric dilatation volvulus (GDV) followed by gastropexy.
Sample Size	203 dogs.
Intervention Details	Case records were reviewed for dogs who had undergone surgical treatment for GDV and who had an open approach to ventral midline gastropexy (n = 329), with 203 dogs having long-term follow-up available. Follow-up was performed with a combination of telephone questionnaire to dog owners, physical examination, and ultrasonographic examination of the gastropexy site.
Study design	Retrospective single-centre case series.
Outcome studied	Outcome and recurrence of GDV.
Main Findings (relevant to PICO question)	91/203 dogs were alive with a median follow-up of 42 months (range 7 to 123 months). 99/203 dogs had died or were euthanised for unrelated reasons with a median follow-up of 31 months (range 2 to 107 months). 13/203 dogs had recurrence of gastric dilatation (GD)/GDV with a median follow-up of 20 months (range 3 to 50 months). 2/13 dogs had recurrence of GDV confirmed surgically, the remaining 11/13 dogs had clinical signs consistent with GD/GDV but responded to medical management therefore it was inconclusive whether these dogs had recurrent GDV.
Limitations	Retrospective study. Absence of a control group. Dropout bias. Wide variation in length of follow-up. Only one gastropexy method was studied. Not all dogs were exposed in a GDV fertile scenario, it is impossible to know if the non-recurrent GDV was due to the gastropexy (if intact) protection or not. Due to limitations in case definition, recurrent GD was also included in case numbers. Due to limitations of follow-up methodology, recurrent GDV could not be conclusively confirmed or excluded in 110 dogs. Genetic and lifestyle bias of sample population from a limited geographical area. Referral hospital bias.

Table note...

**Wacker et al. (1998):** Ultrasonographic evaluation of adhesions induced by incisional gastropexy in 16 dogs **Aim: **To assess the gastropexy site for permanent adhesions in clinical cases.

Population	Dogs presented to the Small Animal Clinic, University of Bern in Switzerland between October 1993 and February 1996, and underwent surgical correction of gastric dilatation volvulus (GDV) followed by gastropexy.
Sample Size	16 dogs.
Intervention Details	Dogs (n = 8) were recruited to the prospective arm of the study and underwent a modified incisional gastropexy. Follow-up was performed via ultrasound exam at 3, 12, and 67 days. For the retrospective arm of the study, case records were reviewed for dogs (n = 28) who had undergone surgical treatment for GDV and who had a modified incisional gastropexy.
Study design	Prospective and retrospective single-centre case series.
Outcome studied	Ultrasonographic integrity of incisional gastropexy.
Main Findings (relevant to PICO question)	8 dogs in prospective group underwent incisional gastropexy. 28 dogs who underwent incisional gastropexy identified in retrospective group. 26/28 dogs survived to discharge. 22/26 dogs had long-term follow-up available. 16/22 dogs were alive at the time of long-term follow-up. 6/22 dogs had died or were euthanised for unrelated reasons. 8/16 dogs were available for ultrasonographic exam. 0/16 dogs (8 from prospective arm and 8 from retrospective arm) had recurrent GDV.
Limitations	Absence of a control group. Dropout bias. Limitations of length of follow-up. Genetic and lifestyle bias of sample population from a limited geographical area. Referral hospital bias.

Table note...

## Appraisal, application and reflection

The PICO question did not apply to the larger population of dogs with risk factors for gastric dilatation volvulus (GDV) undergoing prophylactic gastropexy and was specifically restricted to include only the subset of dogs who have had an episode of GDV. In terms of disease definition, GDV was strictly defined to exclude cases of gastric dilatation (GD) without volvulus because GD, either as a first episode or a recurrent episode, may be managed medically (Przywara et al., 2014).

Eggertsdóttir et al. (1996) reported conducting a non-blinded randomised controlled trial. Recurrence of GDV was reported to be 0/10 (0% after gastropexy and 3/8 dogs (37.5%) without gastropexy. However, on review of the reported methodology, major limitations were identified. Unbalanced (2:1) randomisation was employed and no statistical analysis was reported to show that the control group and treatment group were otherwise equivalent. The method of follow-up was not reported and the possibility of false-positive and false-negative results leading to overestimation and underestimation, respectively, of the true rate of recurrent GDV is possible. This study therefore provides low-quality evidence that recurrent GDV rates are low after gastropexy. The inclusion of a control group provides evidence that gastropexy as an intervention directly reduces recurrence, however methodological limitations of this study are recognised.

Eggertsdóttir et al. (2001) reported conducting a double-blinded randomised controlled trial comparing circumcostal gastropexy to gastrocolopexy. While the comparison was not directly related to the PICO question, the reported recurrence rates of 2/22 dogs (9%) and 4/20 dogs (20%) respectively were relevant. Satisfactory randomisation was reported; however, no statistical analysis was reported to prove equivalence of both groups. This trial was double-blinded, however, methodology of blinding was not clear. In addition, there were limitations in the reporting of the follow up method. Recurrent GDV could not be conclusively excluded in 12/16 dogs with this reliance on remote follow-up, and similarly could not be conclusively diagnosed in 1/3 dogs with this method. As discussed in the previous paragraph, overestimation and underestimation of the true rate of recurrence is possible and impacts the certainty of the reported results.

Two cohort studies (Glickman et al., 1998; Meyer-Lindenburg et al., 1993), one case-control study (Jennings & Butzin, 1992), and eleven case series (Belandria et al., 2009; Belch et al., 2017; Benitez et al., 2013; Formaggini & Degna, 2018; Funkquist, 1979; Leib et al., 1985; Mann et al., 2023; Przywara et al., 2014; Rawlings et al., 2002; Ullmann et al., 2015; Wacker et al., 1998) were appraised.

Losses to follow-up were observed in most studies and follow-up rate, where reported, was reported to have been between 61.7% to 100%. Losses greater than 20% are likely to introduce significant bias (Dettori, 2011) and would have impacted the validity of reported results.

Certain studies (Belandria et al., 2009; Belch et al., 2017; Eggertsdóttir et al., 1996; Funkquist, 1979; Glickman et al., 1998; Mann et al., 2023; Meyer-Lindenburg et al., 1993; Ullmann et al., 2015) were limited by length of follow-up. While there are no objective criteria defining the optimal length of follow-up after GDV and gastropexy, a dog who has had an episode of GDV is likely to have risk factors predisposing to recurrent GDV throughout the rest of the dog’s life. Age has been shown to be a risk factor for this condition (Glickman et al., 1994). In the absence of a longitudinal lifetime study, the appraised studies likely underestimate the true rate of recurrent GDV. Length of follow-up was also not always explicitly reported (Jennings & Butzin, 1992).

Certain studies (Belandria et al., 2009; Belch et al., 2017; Eggertsdóttir et al., 1996; Eggertsdóttir et al., 2001; Formaggini & Degna, 2018; Funkquist, 1979; Glickman et al., 1998; Jennings & Butzin, 1992; Leib et al., 1985; Mann et al., 2023; Przywara et al., 2014; Ullmann et al., 2015) were limited by follow-up methodology. Owner and referring veterinarian reporting of recurrent GDV is imperfect and may introduce bias, which may underestimate the true incidence of recurrent GDV, as discussed above. Dogs who were not alive at the time of follow-up and reported to not have had a recurrent GDV cannot be assumed to have not had recurrent GDV. In the absence of antemortem diagnostics or necropsy evidence, the rate of recurrence would have been underestimated. Similarly, dogs reported to have had recurrent GDV and who were subsequently euthanised without antemortem diagnostics or necropsy evidence cannot be assumed to have had recurrent GDV and the rate of recurrence would have been overestimated. Follow-up via mail survey (Jennings & Butzin, 1992) also risks misclassifying cases with recurrent GDV. Some studies included dogs that died almost immediately (Glickman et al., 1994) in their statistical analysis.

The definition of recurrence varied between studies and some studies (Meyer-Lindenburg et al., 1993; Ullmann et al., 2015) included recurrent GD along with recurrent GDV, which confounded the interpretation of results, as per this Knowledge Summary’s definition.

Benitez et al. (2013) reported no recurrent GDV; however, the sample population of the study also included dogs who received a prophylactic gastropexy and were not relevant to the PICO question.

Marked variation in gastropexy technique was present between studies. Included studies reported the use of the incisional gastropexy, modified incisional gastropexy, circumcostal gastropexy, modified circumcostal gastropexy, modified belt-loop gastropexy, ventral midline gastropexy, appositional gastropexy, stapled gastropexy, modified tube gastropexy, laparoscopic-assisted gastropexy, left fundupexy, and gastrocolopexy. No conclusions can be drawn about the optimal gastropexy technique and this was beyond the scope of this Knowledge Summary.

Of the 16 studies appraised, recurrence of GDV was not a primary outcome in two studies (Rawlings et al., 2002; Wacker et al., 1998). Due to study design and evaluation of a different primary outcome, there were limitations in follow-up length and methodology in both studies, limiting the robustness of their results. In most of the studies, there are important limitations in that not all dogs with no recurrence of GDV were exposed in a GDV fertile scenario. For this reason, it is hard to know if the non-recurrent GDV in these dogs was due to gastropexy (if intact) or not.

Recurrence of GDV after gastropexy, across all studies, was reported to be between 0% and 15.4%. The recurrence rate after gastrocolopexy was reported to be 20% (Eggertsdóttir et al., 2001) and this procedure should not be considered an equivalent to other gastropexy techniques involving the body wall. Given the aforementioned limitations in methodology of follow-up and the likelihood of underestimation of true rate of recurrence, the certainty in the reported recurrence rates is low. Overall, the studies suggest that the recurrence GDV after gastropexy is low to negligible. The low level of the evidence available makes the true recurrence rate less certain. The lack of a standardised method for determining recurrence and a defined time-period for recurrence makes interpretation of the available evidence more challenging. Given the incidence of GDV in clinical practice and the possibility of recurrence, a call for more high-quality, standardised research into this topic is needed.

### Methodology

**Search Strategy:** 

Databases searched and dates covered	CAB Abstracts on OVID Platform covering from 1973 to July 2024 PubMed via the NCBI website covering from 1979 to July 2024
Search terms	CAB Abstracts: (dog or dogs or canine or canines or bitch or bitches or puppy or puppies).mp. or exp dogs/ (gastric dilatation or gastric dilatation volvulus or GDV or gastric torsion or stomach volvulus or bloat).mp. (gastropexy or ((stomach or gastric) and sutur*)).mp. 1 and 2 and 3 Pubmed: (dog OR dogs OR canine OR canines OR bitch OR bitches OR puppy OR puppies) (gastric dilatation OR gastric dilatation volvulus OR GDV OR gastric torsion OR stomach volvulus or bloat) (gastropexy OR ((stomach or gastric) AND sutur*)) 1 and 2 and 3
Date searches performed:	11 July 2024

Table note placeholder

**Exclusion / Inclusion Criteria:** 

Exclusion	Opinion pieces, review articles, articles on GDV or gastropexy but not on recurrence.
Inclusion	Articles that were relevant to the PICO question.

Table note placeholder

**Search Outcome:** 

Database	Number of results	Excluded — opinion pieces or review articles	Excluded — case reports	Excluded — not relevant to the PICO question	Excluded— not on recurrence	Excluded— not accessible	Total relevant papers
CAB Abstracts	155	30	26	14	73	0	12
PubMed	118	13	20	11	61	0	13
Total relevant papers when duplicates removed							16

Table note placeholder

### ORCiD

Daniel Low: https://orcid.org/0000-0003-0215-4997

### Conflict of Interest

The author declares no conflicts of interest.

## Disclaimer

Knowledge Summaries are a peer-reviewed article type which aims to answer a clinical question based on the best available current evidence. It does not override the responsibility of the practitioner. Informed decisions should be made by considering such factors as individual clinical expertise and judgement along with patient’s circumstances and owners’ values. Knowledge Summaries are a resource to help inform and any opinions expressed within the Knowledge Summaries are the author’s own and do not necessarily reflect the view of the RCVS Knowledge. Authors are responsible for the accuracy of the content. While the Editor and Publisher believe that all content herein are in accord with current recommendations and practice at the time of publication, they accept no legal responsibility for any errors or omissions, and make no warranty, express or implied, with respect to material contained within. For further information please refer to our Terms of Use.
